# Characterization of Low-Alcohol Wines Obtained by Post-Fermentative Reverse Osmosis and Vacuum Concentration

**DOI:** 10.3390/foods15020321

**Published:** 2026-01-15

**Authors:** Răzvan Vasile Filimon, Florin Dumitru Bora, Constantin Bogdan Nechita, Marius Niculaua, Cătălin Ioan Zamfir, Roxana Mihaela Filimon, Ancuţa Nechita, Valeriu V. Cotea

**Affiliations:** 1 Research Development Station for Viticulture and Winemaking Iasi, 48 Mihail Sadoveanu Alley, 700490 Iasi, Romania; razvan_f80@yahoo.com (R.V.F.); ancuta.vasile@yahoo.com (A.N.); 2 Faculty of Biology, “Al. I. Cuza” University of Iasi, 11, Carol I Boulevard, 700506 Iasi, Romania; 3 Department of Viticulture and Oenology, Faculty of Horticulture and Business in Rural Development, University of Agricultural Sciences and Veterinary Medicine Cluj-Napoca, 3-5 Mănăștur Street, 400372 Cluj-Napoca, Romania; boraflorindumitru@gmail.com; 4 Laboratory of Chromatography, Advanced Horticultural Research Institute of Transylvania, Faculty of Horticulture and Business in Rural Development, University of Agricultural Sciences and Veterinary Medicine Cluj-Napoca, 3-5 Mănăștur Street, 400372 Cluj-Napoca, Romania; 5 Research Centre for Oenology Iasi, Romanian Academy-Iasi Branch, 9H M. Sadoveanu Alley, 700490 Iasi, Romania; bnechita@gmail.com (C.B.N.); niculaua@acadiasi.ro (M.N.); catalin.zamfir@acadiasi.ro (C.I.Z.); valeriu.cotea@iuls.ro (V.V.C.); 6 Faculty of Horticulture, “Ion Ionescu de la Brad” University of Life Sciences, 3, Mihail Sadoveanu Alley, 700490 Iasi, Romania

**Keywords:** blending, dealcoholization, healthy lifestyle, membrane technology, volatile compounds

## Abstract

In the context of climate change and the general trend toward a healthy lifestyle, reducing the alcoholic strength of wines poses a major challenge for producers. In order to obtain quality low-alcohol wines (LAWs), Muscat Ottonel conventional wine was subjected to reverse osmosis followed by vacuum concentration of the hydroalcoholic permeate (ROVC) or to two-step vacuum concentration (TSVC), with the recovery of aromas as the first alcoholic fraction (F1). Beverages with alcoholic concentrations of 3.50, 5.50, and 8.50% vol. were obtained, with compositional characteristics and sensory properties varying significantly with alcoholic strength and dealcoholization technique applied. ROVC produced wines with organic acids, volatile constituents, extract, and color intensity decreasing progressively with the reduction in alcohol concentration. At similar alcohol concentration, TSVC LAW showed a significantly higher phenolic content, antioxidant activity, volatile compounds (including esters and terpenes), and overall structural balance, maintaining better the typicity of wines. In both processes, reducing alcohol below 5.50% vol. significantly affected the quality and acceptability of the final product. Hierarchical cluster analysis indicated that TSVC LAWs were statistically closer to the conventional wine (control). These findings improve the understanding of how dealcoholization technologies affect the composition of wine, improving product quality, sustainability, and operational efficiency.

## 1. Introduction

Wine benefits from the most rigorous legislation, both in terms of production technologies and quality standards. Although, in accordance with the current legislation, wine must have a minimum acquired alcoholic strength of 8.50% (*v*/*v*), and the alcoholic content of commercial wines is usually much higher. Over time, the alcoholic concentration of wines has evolved progressively, a trend initially attributed to climate change, more specifically, to a gradual increase in air temperatures which led to more important accumulation of carbohydrates in grapes [1]. Later, this trend was also associated with improvements in viticultural practices, the selection of genotypes with a high capacity for sugar accumulation in grapes, and the use of osmotolerant, high-alcohol-producing yeasts for must fermentation [1,2,3].

Excessive alcohol consumption is often linked to increased overall mortality and a higher risk of cancer, but there is evidence indicating that moderate wine consumption may be associated with specific health benefits, providing cardioprotection and neuroprotection and increasing longevity [4,5,6,7]. Globally, an estimated 4.1% of all new cases of cancer in 2020 were attributed to alcohol consumption [8]. In recent years, frequent bans and limitations on the consumption of chemically synthesized additives have made genuine low-alcohol wine an extremely attractive alternative. Most low-alcohol and alcohol-free beverages, except for a few high-quality options, contain numerous additives (sweeteners, flavorings, colorings, etc.), which may pose risks to human health.

In modern nutrition strategies, low-alcohol and non-alcoholic beverages are expected to gradually replace drinks with high alcohol concentrations. From another perspective, the reduction in ethanol in beverages has become essential to enhance their suitability for specific groups of consumers, whether for health, religious, or personal reasons (pregnancy, driving). In these conditions, for producers, diversifying their assortment with wines of reduced alcohol content represents an effective means of capitalizing on grape production. Furthermore, the development of new, high-quality low-alcohol wines at competitive prices enhances both market appeal and producer profitability [9,10].

Besides its negative psychological and physiological effects on human health, ethanol is indispensable for the aging, stability, and organoleptic properties of wine [2,11,12]. From this perspective, low-alcohol wines appear more feasible than alcohol-free wines, representing an intermediate solution that balances producer interests with consumer preferences.

As mentioned in the OIV-OENO 394A-2012 resolution, dealcoholization is the process performed to reduce part or almost all the ethanol content of wines, obtaining viticultural products with low-alcohol content [13]. Although several classifications have been made based on the alcoholic strength, in general, wines can be classified as alcohol-free (<0.5% vol.), low-alcohol (0.5% to 1.2% vol.), reduced-alcohol (1.2% to 6.5% vol.), lower-alcohol (6.5% to 10.5% vol.), and alcoholic wines (>10.5% vol.) [14,15]. Considering that domestic consumption is declining and is now at its lowest level in the last 30 years, and the drop in consumption is highest among younger consumers (who not only tend to consume less, but are more likely to prefer drinks with less or no alcohol), the European Comision proposal 2025/0071 (COD) introduced a revision to existing labeling practices by stipulating that the designation “partially dealcoholised” shall be replaced with the standardized term “reduced-alcohol” for wines with an alcoholic strength above 0.50% vol. and at least 30% below the minimum actual alcoholic strength of the category [16].

To achieve lower alcohol concentrations in wine, several strategies have been proposed, including pre-fermentation, fermentative, and post-fermentation approaches [3,10,11,17,18,19,20]. In addition to preventive methods (short-term strategies) applied only under specific conditions or in certain years, such as during heat waves or drought, the most recommended approaches are those involving the removal of alcohol from finished wines produced from fully mature grapes [2,3,20], considered more effective in preserving the original character of the low-alcohol wines (LAW) (reverse osmosis, pervaporation, or osmotic distillation). The optimal method for ethanol removal from wine depends on multiple factors, including the target ethanol level, production volume, financial investment, and operating costs. Producers must carefully assess these parameters to select the most efficient and cost-effective strategy, while ensuring the preservation of the sensory integrity of LAW.

The membrane processes demonstrate promising results for dealcoholization [21,22]. Nevertheless, the most widely used dealcoholization techniques appear to be vacuum distillation and reverse osmosis [14,23], despite their possible removal of fruity aroma and the potential accentuation of some unpleasant odors [17,24]. Initially applied to must and wine concentration, reverse osmosis (RO) was subsequently extended to the dealcoholization of wine [14,22]. RO is a membrane-based separation process that employs a hydrophilic semi-permeable membrane with an effective pore size of 0.1 to 1 nm, an ultrafiltration method which can be successfully applied to remove a significant hydroalcoholic fraction from wine (permeate), under high pressure and low temperature [17,25,26]. After the removal of alcohol from the hydroalcoholic fraction by distillation or vacuum concentration, the recovered water is subsequently returned to the concentrated wine (retentate). Combined with other energy-saving processes, RO can be a feasible option for wine dealcoholization, although issues like membrane clogging and general lifespan are still being discussed [27,28]. RO offers several advantages over thermal concentration processes, including high selectivity, low energy consumption, reduced operational costs, and room-temperature operation [26]. However, RO is a staged process that requires multiple passes through the membrane to achieve complete dealcoholization of wine, while some aroma compounds (esters and aldehydes), organic acids, and potassium can diffuse through with the alcohol [17].

Vacuum concentration (VC), specifically, low-temperature evaporation, is recommended as an optimal technique as it more effectively preserves the typicity, organoleptic properties, and aromatic profile of the original grapes [15,29]. Various low-pressure and low-temperature evaporators are used, with vacuum concentration operating at temperatures related to those of wine fermentation (<38 °C). Vacuum concentration as a version of vacuum distillation of wine is a thermal process that involves evaporation and condensation of alcohol at low temperature under vacuum conditions [14]. While the underlying physical principle is the same, the two processes differ in their purpose and configuration: vacuum distillation aims to separate and recover volatile components, whereas vacuum concentration focuses on selectively removing volatiles to concentrate the remaining product. Through this vacuum method, most of the alcohol can be removed from wine to levels below 0.50% vol. ethanol. The aromatic fraction, obtained as the first portion of the distillate, may be collected separately and subsequently blended back into the concentrated wine, thereby preserving its sensory profile. As with RO, VC results in the concentration of wine components (phenolic compounds, organic acids, and cations) while eliminating the alcohol [30]. However, this process may result in the loss of volatile compounds, particularly ethyl esters and aliphatic alcohols [14,31]. The OIV’s Resolution OENO-394A-2012 encourages the reduction in alcohol content in wine, partial vacuum evaporation, membrane techniques, and distillation being the main techniques recommended for wine dealcoholization [13].

As previously highlighted, one of the most critical aspects in the process of alcohol reduction is the preservation of wine aromas [14,17,32]. Wine aroma is determined by several groups of volatile compounds—including alcohols, esters, terpenes, pyrazines, acids, phenols, aldehydes, and varietal thiols—their type and concentration exerting a significant influence on the overall aromatic profile of the wine. Whether originating from varietal, fermentation, or aging processes, wine aromas depend on the concentration of individual volatile components. If, after dealcoholization, the concentration of a compound is too low, its contribution to the overall aroma profile will be diminished; conversely, if its concentration is too high, it may mask the perception of other compounds, thereby compromising the balance and complexity of the wine’s aroma [33]. Recent studies reported that the dealcoholization method significantly affects the wine’s volatile profile and sensory characteristics, and depending on the technique used, the degree of alcohol reduction and initial wine composition [14,23,32]. It is therefore necessary to explore new processing technologies aimed at improving the aroma and sensory quality of LAW.

From another perspective, wines with a pronounced aromatic profile are more likely to yield pleasant LAW. Muscat Ottonel (*Vitis vinifera* L.; *Vitis* International Variety Catalogue no. 8243) is an aromatic white grape variety of French origin that spread throughout the vineyards of Central and Eastern Europe, in Romania occupying a total area of about 8000 hectares [34,35]. The Muscat Ottonel variety is highly popular in Romania, where it benefits from very favorable climatic conditions. The resulting white wines are characterized by delicate floral and fruity notes (typical of Muscat), with alcoholic strength often exceeding 12% vol. [36,37]. Despite its widespread use in aromatic wine production, Muscat Ottonel has been scarcely investigated in the context of post-fermentative dealcoholization, particularly with respect to the preservation of its characteristic aroma profile. Considering that the aroma and taste of wine are critical factors for consumer acceptance, the current study aimed at physico-chemical characterization, including changes in phenolic content, main aroma constituents, organic acid profile, chromatic parameters, and sensory attributes of low-alcohol wines (3.50–8.50% vol.) obtained through reverse osmosis followed by vacuum concentration (ROVC) or two-step vacuum concentration (TSVC). The findings of the current study highlight techniques that enhance operational efficiency while maintaining compliance with quality standards, and provide a deeper understanding of compositional changes associated with alcohol removal in wine. These insights are particularly relevant for small and medium-sized winemakers seeking to adapt to global market challenges and meet evolving consumer demands.

## 2. Materials and Methods

### 2.1. Biological Material and Winemaking Procedure

White grapes of the Muscat Ottonel variety (*Vitis vinifera* L.), grafted on the hybrid rootstock Kober 5 BB (*V. berlandieri* Planch. × *V. riparia* Michx.), were harvested at full maturity (constant berry weight and sugars/acidity ratio) from the 38-year old unirrigated plantations of the Research-Development Station for Viticulture and Winemaking Iasi, Copou-Iasi wine-growing centre, NE of Romania (47°12′18″ N, 27°32′03″ E). After transport to the winemaking station, grapes were crushed and destemmed in the industrial winemaking system, under minimum SO_2_ protection (40 mg/L). Following maceration in rotary tanks (8 h; Viazym^®^ MP, Martin Vialatte, Magenta, France) and pressing (pneumatic press model F22, Puleo, Marsala, Italy), the assembled must was clarified by natural settling (12 h) and racking, and then subjected to alcoholic fermentation (Zymasil Bayanus^®^, AEB, Kaysersberg-Vignoble, France; *Saccharomyces cerevisiae* var. *bayanus*), at 18 ± 1 °C, in stainless steel tanks. The raw wine was clarified through settling, racking, fining (Spherobent Super^®^, Laviosa, Livorno, Italy; 35 g/hL), and filtration (Clarcel^®^ Kieselguhr CBL, Chemviron, Rueil-Malmaison, France; 50 g/hL). Apart from a protective sulphite treatment (up to 60 mg/L total SO_2_), no other treatment was applied to the wine.

For an overall picture of the terroir, the Iasi vineyard is characterized by a temperate-continental climate with excessive nuances, marked by pronounced seasonal contrasts, including harsh and dry winters and hot, often arid summers. The plot is located at an altitude of 191 m, on a slight slope (3%) with southern exposure. In the Copou-Iași wine-growing centre, during the period 1971–2020, the multiannual averages indicate a mean temperature of 9.89 °C and annual precipitation of 574.70 mm [1]. In the grape harvest year (2022), the average annual temperature was 11.3 °C, with an absolute maximum of 36.5 °C in August and an absolute minimum of −11.3 °C in January. Annual precipitation totaled 416.8 mm, of which 295.8 mm during the vegetation period (April–September). The year was characterized by a high sunshine duration of 2032 h.

### 2.2. Reverse Osmosis-Vacuum Concentration (ROVC)

A portion of the clarified wine (300 L) was transferred into a thermostatic stainless-steel tank (17 ± 1 °C) connected to a fully automated reverse osmosis unit Flavy ML 2 (Bucher Vaslin, Chalonnes-sur-Loire, France) equipped with a series of two semipermeable polyether sulfone (PES) membranes, pore size of 1 nm. The flow rate was adjusted so that the working pressure used, multiplied by the temperature of the wine passing through the osmosis membranes, does not exceed the value of 1200 cumulative units. The normal operating pressure was 65 bar, and to the exit from the osmosis membranes, the retentate was quantitatively passed through a heat exchanger to maintain a constant working temperature of 17 ± 2 °C, at a flow rate of about 850 L/h.

**Figure 1:**
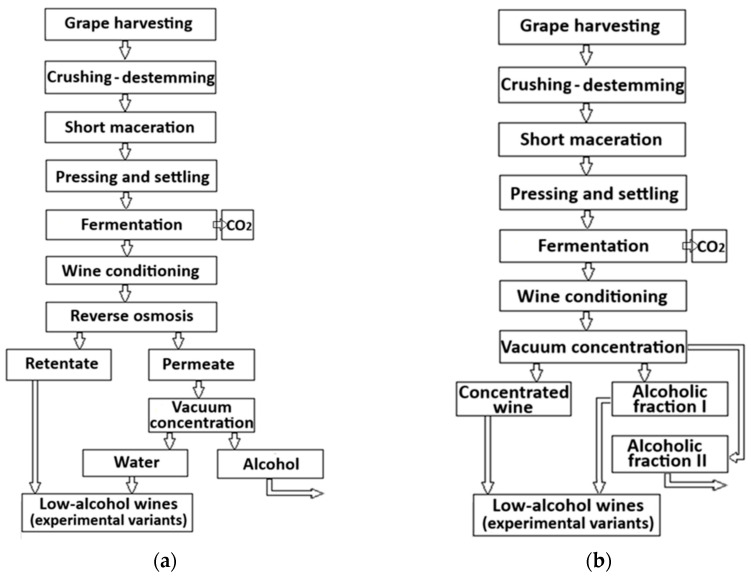
Technological schemes for obtaining experimental low-alcohol beverages: (**a**) reverse osmosis-vacuum concentration and (**b**) two-step vacuum concentration.

For the separation of the alcoholic fraction from the hydroalcoholic permeate by distillation, the vacuum concentration process was used. Thus, small portions of permeate were loaded into a rotary evaporator (HS-2005V, Hahnshin, Bucheon, Republic of Korea), equipped with water bath thermostated at 38 ± 1 °C and a ME 2 NT vacuum pump (Vacuubrand, Wertheim, Germany) with a regulator valve for vacuum (<90 mbar), which ensured the rapid evaporation of the alcoholic fraction, at a rotation speed of 120 RPM, condensed at 7 ± 1 °C and collected continuously during the process. After dealcoholization of the hydroalcoholic permeate (0.45–0.55% vol.), the alcohol-free osmosis water was blended by calculation with the retentate (concentrated wine), in order to obtain three low-alcoholic beverages, with 3.50, 5.50, and 8.50% vol. alcohol (Figure 1a).

### 2.3. Two-Step Vacuum Concentration (TSVC)

The vacuum dealcoholization process applied followed the methodology described by Kumar et al. [38], with some modifications. Small portions of the clarified wine (up to a final volume of 10 L) were loaded into the rotary evaporator. The vacuum pressure was maintained at 90 mbar, with a water bath temperature initially set at 30 ± 1 °C and a flask rotation speed of 70 rpm. The condensation temperature was kept constant at 7 ± 1 °C throughout the experiment. After separating approximately 10% of the wine volume (step 1), containing the most important proportion of volatile aroma compounds (F1), the water bath temperature was increased to 38 ± 1 °C until the wine reached an alcohol content below 0.50% vol (step 2). Fraction F1 was collected separately in glass containers with screw cap and stored in the refrigerator until use (6 ± 1 °C). The second alcoholic fraction was collected and removed from the experiment (F2). To reach an alcoholic concentration < 0.50% vol., a percentage of about 30% of the initial volume of the wine was evaporated. After dealcoholization, the concentrated wine was blended by calculation with different volumes of F1, obtaining three low-alcoholic wines with 3.50, 5.50, and 8.50% vol. alcohol (Figure 1b). These concentrations were selected to cover a practical range of low-alcohol wines of interest to both producers and consumers while remaining sensorially acceptable.

### 2.4. Physico-Chemical Characteristics of Wine

All LAWs and the control wine (11.80% vol.) were analyzed in terms of alcoholic concentration (% vol.), volatile acidity (g/L acetic acid) (oenological distiller-extractor DE 2000, Dujardin-Salleron, Noizay, France), total acidity (g/L tartaric acid), free and total SO_2_ (by titration, mg/L), relative density, non-reducing extract (g/L), pH (inoLab Level 1 pH meter, WTW, Weilheim, Germany), and reducing sugars (g/L), according to the Compendium of International Methods of Wine and Must Analysis [39].

Free volatile terpenes (FVT) and precursor terpenes (PVT) were evaluated by distillation (distiller-extractor DE 2000, Dujardin-Salleron, Noizay, France), using the method proposed by Dimitriadis and Williams [40], the results being expressed as μg/L as linalool equivalent (y = 0.0165x + 0.044; R^2^ = 0.9991) (analytical standard; Supelco, Darmstadt, Germany). All spectral determinations were performed on a UV-vis Specord 200 plus spectrophotometer (Analytik Jena, Jena, Germany).

### 2.5. Phenolic Composition, Antioxidant Activity, and Chromatic Parameters

Total phenolic content (TPC) was determined using Folin–Ciocalteu reagent (Scharlab, Barcelona, Spain), according to OIV-MA-AS2-10 method [39]. Gallic acid (g GAE/L) was used as a standard (y = 1.2114x + 0.0118; R^2^ = 0.9910). Optical density at 280 nm was measured based on the characteristic absorption of the benzene rings of most phenols at this wavelength and reported as total phenolic index (TPI) [41]. The Bate-Smith colorimetric method based on the hydrolysis reaction of condensed tannins in a heated acidic medium was used for the estimation of proanthocyanidins, multiplying the difference in absorbance at 550 nm by 19.33 (g/L as catechin equivalent) [42]. DPPH (2,2-diphenyl-1-picrylhydrazyl; Alfa Aesar, Karlsruhe, Germany) free radical scavenging activity was measured according to the procedure proposed by Brand-Williams et al. [43]. The reaction mixture containing 0.1 mL of wine and 3.9 mL of 2.36% DPPH in 96% ethanol solution was incubated at 37 °C for 30 min, and the reduction in absorbance was measured at 517 nm. The antioxidant activity was reported as a percentage of scavenged DPPH* (%) = ((Abs control − Abs test)/Abs control) × 100. Ascorbic acid and gallic acid (0–100 μg/mL) were used as positive controls, according to protocol presented by Filimon et al. [44].

Chromatic parameters of wines (CI—color intensity, H—color hue, and the proportions of yellow, red, and blue) were assessed by determining the optical density (OD) of the samples at 420, 520, and 620 nm, and subsequent calculation: CI = OD420 nm + OD520 nm + OD620 nm; H = OD420 nm/OD520 nm; the percentage of yellow (%Y = OD420 nm/CI·100), red (%R = OD520 nm/CI·100), and blue (%B = OD620 nm/CI·100) [45].

### 2.6. Volatile Compounds Analysis

Volatile compounds were identified and quantified by gas chromatography, using an Agilent 7890A gas-chromatograph (Agilent Technologies, Lexington, KY, USA), with a sensitive flame ionization detector (FID). The determination of volatile compounds in wines was conducted according to the method OIV-MA-AS315-27:2018 [46], with some modifications. The method was adapted for headspace analysis. Volume injected: 1500 µL (HS-2.5 mL syringe), the sample incubation time 900 s, at an incubation temperature of 85 °C. The volatile compounds were separated using a Phenomenex FFAP (Phenomenex Inc., Torrance, CA, USA) polar-phase column, with length of 48.5 m, internal diameter of 0.32 mm, and film thickness of 0.45 µm. The calibration was performed by injecting the calibration solutions (50–250 mg/L) before each series of analysis. Each compound was identified and quantified based on the calibration curves of standard compounds, with coefficients of determination ranging between 0.99129 and 0.99896.

### 2.7. Determination of the Organic Acid Profile

Organic acids were separated and simultaneously determined by high performance liquid chromatography—diode array detector (HPLC-DAD), using a Prominence LC series 20 system (Shimadzu Scientific Instruments, Canby, OR, USA) and the OIV modified procedure OIV-MA-AS313-04 [39]). The HPLC system includes a five-channel degasser (DGU-20A5R), two quaternary pumps (LC-20AD), an auto-injector (SIL-20ACHT), thermostatically controlled column oven (CTO-20AC), and diode array detector (Nexera X2 SPD-M30A), coupled to a desktop PC system via LabSolution v. 5.6 software. A total of 5 μL of sample or reference were injected into the HPLC system. Oxalic, tartaric, malic, lactic, shikimic, acetic, citric, succinic, and fumaric acids were determined using two chromatographic columns: a Phenomenex Kinetex C18 column (Phenomenex Inc., Torrance, CA, USA) (50 mm × 4.6 mm, 5 µm superficially porous particles, 100 Å pore size) and a YMC TriArt C18 column (YMC, Allentown, PA, USA (250 mm × 4.6 mm, 5 µm solid-core spherical particles, 120 Å pore size). The mobile phase consisted of a 9 mM methanesulfonic acid (MSA) solution (pH 2.3, conductivity 2.75 mS/cm). The elution flow rate was set to 1.5 mL/min, with the first column maintained at 15 °C and the second at 45 °C. Detection was performed at 210 nm for most organic acids, while shikimic acid was quantified at 215 nm. All samples were filtered through 0.45 µm regenerated cellulose (RC) membrane filters prior to analysis. All reagents and standards used were of analytical grade (Merck, Darmstadt, Germany).

### 2.8. Basic Sensory Profile of Low-Alcohol Wines

Sensory analysis of the obtained LAWs was carried out by a panel of nine trained tasters (4 men and 5 women) consisting of PhD students, researchers, and specialists in oenology. Prior to evaluation, panelists underwent training sessions to familiarize them with the sensory descriptors used and the scoring procedure. Samples were evaluated under controlled sensory laboratory conditions, following standard sensory analysis guidelines. Assessors scored 15 attributes (green/vegetal, mineral, ripe fruit, exotic fruit, dried fruit, muscat, acidic, sweet, phenolic, full-bodied, taste persistence, clarity, color intensity, varietal typicity, and structural balance) using a structured 10-point scale, where 1 represented the lowest intensity or poorest quality and 10 the highest intensity or best quality. Each participant received 20 mL of the beverage, served in glass cups at a temperature between 8 and 10 °C. Sample presentation order was randomized. To neutralize residual flavors between samples, tasters were provided with square pieces of white bread and still water. All samples were analyzed in triplicate, and results are presented as descriptive statistics (mean ± standard deviation). Sensory data were recorded and processed using Microsoft Excel^®^ 2023 (Microsoft Corporation, Redmond, WA, USA).

### 2.9. Statistical Procedures

Data were reported as the mean of minimum three replicates with standard deviation (±). Analysis of variance (ANOVA test) was initiated to investigate significant differences between data in XLSTAT^®^ Cloud, within Microsoft Excel^®^ 2023 software (Microsoft Corporation, Redmond, WA, USA). The method used to discriminate among the means was Tukey’s multiple range test at 95% confidence level. *p* values lower than 0.05 (*p* < 0.05) were considered to be significant. Different letters indicate significant differences between data. Regression analysis was performed to look for relationships between data. Principal component analysis (correlation type) with agglomerative hierarchical clustering (single-linkage; Euclidian distance) and histograms were performed to investigate data group formation (XLSTAT Cloud for Microsoft Excel^®^). Spider plots of sensory profiles were generated with Microsoft^®^ Excel 2023 software.

## 3. Results and Discussion

### 3.1. Obtaining LAW by the ROVC Process

After separating 50% of the Muscat Ottonel (MO) wine, the hydroalcoholic fraction obtained as permeate had an alcoholic concentration of 10.72% vol., while the retentate showed an alcoholic concentration of 12.85% vol. (Table 1). Thus, for 300 L of wine with 11.8% vol. alcohol, separated 50:50 by reverse osmosis, the mass balance was maintained, with only minimal alcohol losses during processing (~0.1%). However, all components of the wine matrix (retentate) were concentrated after RO process. Thus, the retentate reached a total acidity by about 60% higher (7.78 g/L as tartaric acid) compared to the initial wine (4.82 g/L as tartaric acid), an almost double concentration of reducing sugars (1.76 g/L) and SO_2_ (total and free). As frequently noted in the literature, reverse osmosis (RO) was initially developed as a technique for concentrating wines, with the purpose of increasing alcohol content and adjusting chemical composition [26].

**Figure 2:**
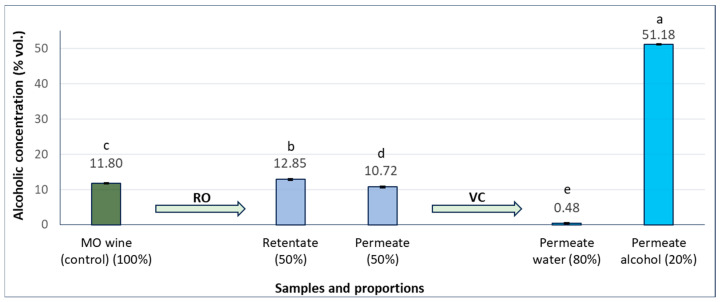
Changes in alcoholic concentration (% vol.) of Muscat Ottonel (MO) wine fractions obtained by reverse osmosis (RO) and subsequent vacuum concentration (VC). Different letters indicate significant differences between the mean values in Tukey’s multiple range test.

The permeate was subsequently subjected to vacuum concentration, yielding two fractions: an alcoholic fraction (approximately 20% of the permeate volume) containing about 51% vol. ethanol, and an aqueous fraction (approximately 80% of the permeate volume) with less than 0.50% vol. ethanol. Variations in the alcoholic concentration of the fractions obtained throughout the RO process are shown in Figure 2.

The osmosis water (<0.50% vol. alcohol) was blended by calculation with the concentrated wine (retentate), obtaining three experimental LAWs with alcoholic concentrations of 3.50, 5.50, and 8.50% vol.

### 3.2. Obtaining LAW by the TSVC Process

First alcoholic fraction (F1), which contained most of the volatile compounds, showed a mean alcoholic concentration of 42.60% vol. The second alcoholic fraction (F2), obtained during the continuation of the dealcoholization process and corresponding to 30% of the initial wine volume, exhibited a mean alcoholic strength of 24.10% vol. This fraction was excluded from the study and was not considered for subsequent blending trials. The discarded alcoholic fraction was recoverable and could be further concentrated to achieve higher ethanol purity, enabling potential applications in compound extraction or in the production of fortified alcoholic beverages. Subsequently, the F1 fraction was blended in calculated proportions with different volumes of concentrated wine, resulting in three experimental low-alcohol wines (LAWs) with alcoholic strengths of 3.50%, 5.50%, and 8.50% vol.

**Figure 3:**
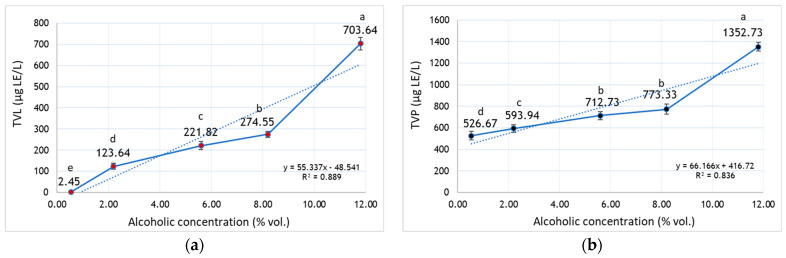
The dynamics of the extraction of (**a**) free volatile terpenes—TVL and (**b**) precursors—TVP during the vacuum concentration of Muscat Ottonel wine. Note: LE—linalool equivalent. Error bars represent standard deviation (±) (*n* = 3). Different letters indicate significant differences between the mean values in Tukey’s multiple range test (*p* < 0.05).

Terpenoids are the main class of odoriferous substances in *V. vinifera* L. grapes, in both forms free or combined as glycoside (precursors of aromas), being particularly responsible for the characteristic aroma of Muscat grapes, musts, and wines [47,48]. To assess the dynamics of aroma compound extraction in parallel with ethanol removal by vacuum concentration (VC), MO wine was progressively concentrated, and the levels of free volatile terpenes (TVL) as well as terpene precursors (TVP) were quantified throughout the process. Distinct patterns in the behavior of free terpenes and their precursors were observed during wine VC. Initially, MO wine showed elevated concentrations of TVL (703.64 µg LE/L) and TVP (1352.73 µg LE/L), as determined by steam distillation followed by vanillin reaction. Reduction in the alcoholic strength from 11.80 to 8.50% vol. through VC resulted in a pronounced decline in free terpene content, with losses of up to 60% (Figure 3a), whereas terpene precursor compounds decreased to a lesser extent, by approximately 40% (Figure 3b). Following vacuum concentration of the wine to <0.50% vol. alcohol, free terpenes were reduced to trace levels, whereas terpene precursors remained at comparatively higher levels, exceeding 500 µg LE/L. A strong positive correlation was observed between TVP and TVL contents, with both decreasing proportionally to the extent of vacuum concentration (r^2^ = 0.9609). According to the results reported by Stoica et al. [49]), the content of free volatile terpenes (TVL) in Muscat grape must ranged between 985 and 2190 µg LE/L, depending on maceration duration, whereas terpene precursor levels (TVP) consistently exceeded 2200 µg LE/L.

The results obtained through vacuum dealcoholization are consistent with those reported by Petrozziello et al. [50], who demonstrated that, in white wine dealcoholized by vacuum distillation from 10.79 to 3.36% vol., most volatile compounds were no longer detectable or quantifiable. To minimize the loss of highly volatile compounds, which are removed in considerable amounts during the initial stages of the dealcoholization process, the first alcoholic fraction, enriched in aroma constituents, was collected and subsequently recombined with the concentrated wine in calculated proportions, yielding experimental wines with various alcoholic concentrations.

### 3.3. Physico-Chemical Characterization of LAW Obtained by ROVC and TSVC

As originally designed, the alcoholic strength of the experimental beverages obtained by the two tested procedures was 3.50 (V1-3.5), 5.50 (V2-5.5), and 8.50 (V3-8.5) % vol. In the case of ROVC, the RO initially concentrated the wine (retentate), while subsequent dilution with osmosis water, derived from alcohol evaporation by VC, led to a proportional reduction across all classes of compounds (Table 2). Thus, the wine subjected to the highest dilution (V1-3.5) exhibited a significantly higher density (volumetric mass) (0.9969 g/cm^3^) than both the initial wine (0.9911 g/cm^3^) and the higher-alcohol variants, while its total acidity and non-reducing dry extract were significantly reduced as a consequence of water addition. By reducing the amount of water added and thereby maintaining a higher alcoholic strength in the ROVC-derived beverages (5.50–8.50% vol.), a balance in total acidity was achieved. At the same time, significantly elevated levels of volatile acidity (0.58–0.82 g/L acetic acid) and total SO_2_ (<89 mg/L) were recorded, in comparison with both the original wine and the lower-alcoholic variants (Table 2). However, the volatile acidity values remained well below the limits stipulated by EU legislation for white wines, 18 mEq/L or 1.08 g/L acetic acid equivalent [51]. Although TSVC wines showed increased volatile acidity, the values remained within generally accepted sensory thresholds for wine (0.6–0.9 g/L acetic acid) and did not result in detectable off-flavors, indicating acceptable sensory quality under the conditions tested.

In the TSVC procedure, the addition of small volumes of fraction F1 (noted for its aromatic richness) to the alcohol-free concentrated wine resulted in a compositional profile of the LAW that contrasted with that obtained by the ROVC method. Thus, the wines with the lowest alcohol concentration (V1-3.5% vol.) showed the highest total acidity (5.47 g/L expressed as tartaric acid), density at 20 °C (1.0030 g/cm^3^) and non-reducing extract (24.29 g/L), and the lowest pH (3.40). Moreover, with increasing alcoholic strength, the total acidity was slightly reduced due to dilution with small portions of fraction F1. In contrast, volatile acidity increased following the addition of the volatile fraction, reaching values of 0.96 g/L acetic acid at an alcoholic concentration of 8.50% vol. (V3-8.5). Italiano et al. [52] highlighted that thermal methods, such as vacuum distillation of wine, effectively concentrate key compounds, including total phenols and minerals, through ethanol removal and the resulting concentration effect. In contrast, membrane-based techniques, particularly reverse osmosis (RO), provide a gentler approach, allowing for better preservation of essential wine parameters. Excluding alcoholic strength, the wine samples obtained through both processes exhibited physico-chemical parameters that complied with the legal requirements for white wines [53]. However, the total acidity of V1-3.5 sample (3.50% vol. alcohol) produced by ROVC (2.55 g/L as tartaric acid) was slightly below the legal threshold (>3.50 g/L as tartaric acid), indicating the need for corrective acidity treatments.

### 3.4. Total Phenolic Content and Antioxidant Capacity of LAW

Phenolic compounds are complex molecules that occur in both red and white wine. Although in white wine phenolic compounds appear in much lower concentrations, they are important contributors to the appearance, antioxidant capacity, and sensory aspects of the wine [54]. The total phenolic content (TPC) of LAW obtained by ROVC varied significantly in relation to their alcoholic concentration (Table 3). ROVC V3-8.5 variant (8.50% vol. alcohol) showed the highest phenolic concentration (0.41 g GAE/L), even higher than the initial wine, due to lower dilution with osmosis water. Increasing the volume of osmosis water added to the retentate resulted in a physical dilution of phenolic compounds, a trend consistent with other classes of wine constituents. This pattern was likewise reflected in the optical density measurements of the samples at 280 nm. However, not all phenolic compounds exhibit their maximum absorption at 280 nm [55].

Proanthocyanidins are condensed tannins with various pharmacological properties, recognized as important qualitative factors in wine due to their role in astringency, bitterness, and color stability [56,57]. LAW obtained through ROVC exhibited reduced proanthocyanidin concentrations, with the highest value recorded in sample V3-8.5, of 0.16 g CE/L, exceeding that of the control wine (0.16 g CE/L). When the alcoholic strength was reduced to 3.50% vol. (V1), the concentrations of phenolic compounds and proanthocyanidins decreased by more than 50% (0.19 GAE/L and 0.04 g CE/L, respectively), remaining within the range reported in the literature for white wines [56].

A comparative evaluation of the two processes revealed that the phenolic content of LAW obtained by TSVC (0.39–0.44 g GAE/L) was consistently higher than that of wines produced by ROVC (0.19–0.41 g GAE/L). In both cases, the differences relative to the control (initial wine) were statistically significant, with the exception of the TSVC V3-8.5 variant, which most closely resembled the conventional wine in terms of phenolic composition. A similar pattern was observed for proanthocyanidin concentrations.

The assessment of antioxidant activity in wine is particularly relevant, as it reflects its potential contribution to reducing oxidative stress and lowering the risk of chronic diseases. In general, white wines exhibit significantly lower phenolic concentrations and antioxidant capacity compared to red wines [58]. In LAW obtained through ROVC, antioxidant activity decreased progressively with the reduction in alcoholic strength (Table 3). Specifically, in the ROVC V3-8.5 sample (8.50% vol. alcohol), DPPH radical scavenging reached 61.06%, whereas in the V2-5.5 sample (5.50% vol. alcohol) the value decreased to 38.92%, and further to 30.01% in the V1-3.5 variant (3.50% vol. alcohol). This represents approximately half the antioxidant activity measured in the control wine (58.69%). On the other hand, the application of the TSVC process resulted in significantly higher antioxidant activity in wines with reduced alcoholic strength (3.50–5.50% vol.), compared to the control wine (>60% scavenged DPPH).

**Figure 4:**
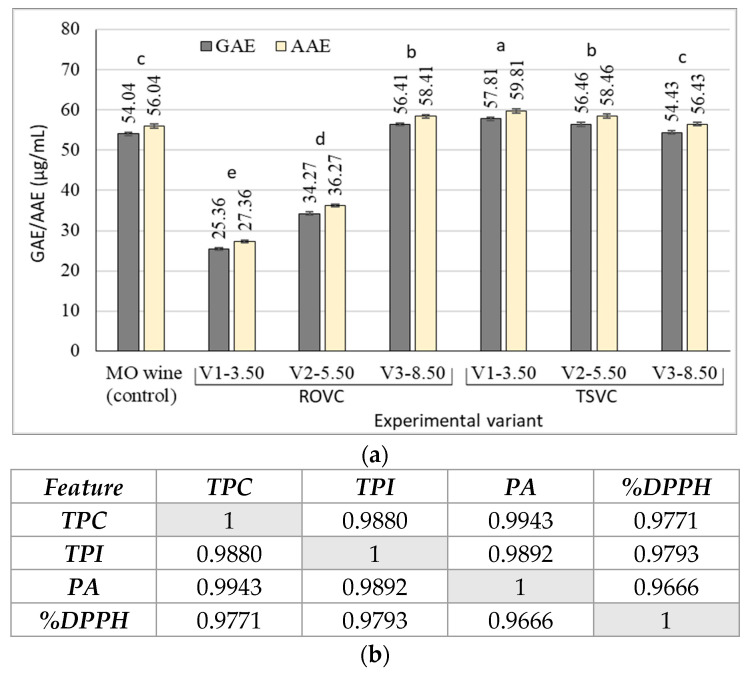
Antioxidant activity of the experimental low-alcohol wines (**a**) and the correlation with the phenolic content (**b**). Note: ROVC—reverse osmosis-vacuum concentration; TSVC—two-step vacuum concentration; GAE—gallic acid equivalent; AAE—ascorbic acid equivalent; TPC—total phenolic content; TPI—total polyphenolic index (OD at 280 nm); PA—proanthocyanidins; %DPPH—% scavenged DPPH. Error bars indicate standard deviation (±) (*n* = 3). Different letters indicate significant differences between the mean values in Tukey’s multiple range test (*p* < 0.05).

Equating the antioxidant activity of LAW with standard compounds such as gallic acid or ascorbic acid revealed that the highest values were obtained in wines produced by TSVC, ranging between 54.43 and 57.81 µg/mL gallic acid equivalent (GAE), and between 56.43 and 59.81 µg/mL ascorbic acid equivalent (AAE), respectively (Figure 4a). These values, together with those recorded for the ROVC V3-8.5 variant (56.41 µg GAE/mL; 56.04 µg AAE/mL), are very close to those of the initial wine (control) and comparable to values reported for other Romanian white wines, which generally range between 31 and 65 µg GAE/mL [59].

The antioxidant activity of LAWs was directly correlated with their phenolic content (Figure 4b), most notably with total phenolic compounds (r^2^ = 0.9771) and to a slightly lesser extent with proanthocyanidins (r^2^ = 0.9666). This correlation underscores the role of phenolic compounds in enhancing the functional potential of wines. Similar strong positive associations between antioxidant activity and different classes of phenolics have previously been reported in white musts and wines, highlighting the contribution of these compounds to the health-promoting value of wine [58,59].

### 3.5. Chromatic Parameters of Wines

Color represents one of the most important attributes of wines, serving as a fundamental criterion for their identification and consumer acceptability. Together with clarity, color is consistently associated with wine quality and safety, making the study of chromatic parameters essential for the comprehensive evaluation of wine. Chromatic parameters of LAWs obtained through ROVC and TSVC were calculated based on the interpretation of their optical density at 420, 520, and 620 nm, as presented in Table 4. For all low-alcohol beverages obtained, the yellow color component contributed the most to color formation (>68%), with red (19–23%) and blue (2–9%) colors participating in lower proportions. However, the initial Muscat Ottonel wine (control) showed the highest proportion of yellow (%Y = 78.04%) and the lowest proportion of red (%R = 18.97%). In LAW samples obtained by reverse osmosis (ROVC), the proportion of yellow (%Y) was lower due to dilution, with values decreasing significantly with the reduction in alcohol concentration by osmosis water addition. If in the case of the yellow color, the values of low-alcohol wines were lower compared to the initial wine (control), by up to 10–12%, and the blue color contribution was up to 3.5 times higher compared to the values registered for the initial wine.

Color intensity (CI) was calculated as the sum of absorbances at 420, 520, and 620 nm, providing an estimate of the overall strength of wine color. In addition, the ratio between absorbance at 420 nm and 520 nm (OD_420_/OD_520_) was used to determine the color hue (H), which reflects the shift in wine color toward orange tones [60]. In wines obtained through ROVC, both color intensity (CI) and hue (H) decreased significantly with the reduction in alcoholic strength (Table 5). The ROVC sample with the lowest alcohol concentration (V1-3.5) exhibited a reduced color intensity (CI = 0.02) and hue (H = 3.02), and a lower contribution of yellow (69.74%) and blue (7.52%) components. In parallel, a significant increase in the proportion of red hues (23.01%) was observed.

LAW obtained through TSVC showed an inverse variation in chromatic parameters compared to ROVC, reflecting the distinct method of production. In the case of ROVC, the addition of osmosis water is intended to reduce the alcoholic strength of the concentrated wine, diluting the wine matrix. By contrast, in TSVC, the addition of small amounts of the alcoholic–aromatic fraction serves to increase the alcoholic strength of the concentrated wine while simultaneously restoring its aromatic profile. In TSVC samples, progressive dilution of the concentrated wine (decreasing ethanol amount) resulted in a reduction in the yellow (%Y) and blue (%B) chromatic components, accompanied by a slight increase in red hues (%R). This shift toward warmer tones (amber/orange) can be attributed to concentration effects and the oxidation of phenolic compounds, as previously demonstrated [23,32]. For TSVC low-alcohol wines, no significant differences in color intensity (CI) were observed among the variants. The highest CI value was recorded in the wine with the lowest alcohol concentration (0.10), a trend that was also reflected in the color hue (tonality), which reached 3.41. Overall, reverse osmosis-vacuum concentration (ROVC) was found to affect the color of LAW to a much greater extent than the two-step vacuum concentration (TSVC) at similar alcohol concentrations, resulting in perceptible alterations in their chromatic composition. However, the color intensity (CI) of Muscat Ottonel LAW (<0.11) remained lower than the values typically reported for wines produced from white grape varieties, which generally range between 0.3 and 1.5 [61].

### 3.6. Organic Acid Profile of LAW

Acids are part of the fundamental structure of wine [62]. Organic acids play a critical role in wine quality, as they influence its sensory attributes, chemical stability, chromatic expression, and aging potential [63,64]. They influence freshness, balance, color retention, and microbial safety, making them critical to both winemaking and sensory experience [65,66]. Also, the type and concentration of organic acids directly influence the pH of the wine. Most organic acids in wine originate from grapes, while only a minor fraction is produced during alcoholic and malolactic fermentations through the metabolic activity of yeasts and lactic acid bacteria [66,67]. Both the ROVC and TSVC techniques exerted a significant impact on the organic acid profile of the resulting low-alcohol wines. Eight of the most relevant organic acids were identified and quantified by HPLC in LAW produced through the two procedures (Table 5).

In ROVC LAW, the concentration of the main acids decreased in parallel with the reduction in alcoholic strength, with statistically significant differences observed between the samples. Thus, in the case of the V3-8.5 variant obtained by ROVC, the concentrations of the main organic acids were the highest, remaining close to those of the initial wine (control). For malic and citric acids, the differences between the V3-8.5 sample and the control wine were statistically non-significant. Tartaric acid, as the main organic acid naturally occurring in grapes and, therefore, a vital contributor to the wine acidity [62], was found in relatively low concentrations in ROVC LAW, ranging from 1.16 (V1) to 3.81 (V3). Muscat Ottonel grapes are usually deficient in acidity, presenting low concentrations of organic acids upon ripening [68], often requiring acidity corrections applied to the must or wine. In Europe, Regulation (EU) No. 1308/2013 authorizes the use of L-tartaric, L-malic/DL-malic, and lactic acids for wine acidification, with the maximum permissible increase in total acidity set at 1.5 g/L for grape must and 2.5 g/L for wine, as tartaric acid [69].

Acetic acid, the principal constituent of volatile acidity in wine, is formed as a by-product of yeast metabolism during alcoholic fermentation or through the activity of various spoilage microorganisms [70]. In wines concentrated by reverse osmosis and subsequently blended with osmosis water to an alcoholic strength of 8.50% vol. (ROVC V3-8.5), acetic acid content increased by approximately 50% compared to the control wine, reaching 0.78 g/L. As the main component of volatile acidity, acetic acid is typically detectable within the range of 0.6–0.9 g/L [71].

A phenomenon observed in reverse osmosis-concentrated wine (retentate) was the precipitation of tartaric acid salts, resulting from a disturbance in the wine matrix associated with the alcoholic fraction. Unlike alcoholic wine, where ethanol significantly reduces the solubility of potassium bitartrate (KHT) crystals, tartrate precipitation in non-alcoholic beverages is mainly governed by low temperature, elevated pH, and the concentration of the acids [72].

Succinic acid is a natural by-product of alcoholic fermentation, the main non-volatile carboxylic acid produced by yeast during the early stages of alcoholic fermentation [73,74]. Succinic acid imparts a harsh, sour taste and exerts a marked influence on the pH, sensory profile, and overall stability of wine [75]. In LAW produced by the ROVC process, succinic acid concentrations (425.71–490.31 mg/L) were lower than in the control wine (539.58 mg/L), with the sample containing 3.50% vol. alcohol (V1-3.5), showing the lowest values.

LAW obtained through the staged vacuum concentration process (TSVC) displayed an inverse trend in organic acid levels compared to those produced by ROVC, highlighting differences in the production process. Samples with lower alcoholic strength (V1-3.5% vol.) contained the highest concentrations of organic acids. However, similar to the ROVC process, significant variations in organic acid content were also noted among the TSVC samples. A particular case was that of acetic acid, which under vacuum, partially passed into the evaporated fraction. Thus, acetic acid was added in TSVC wines with the addition of the alcoholic–aromatic fraction F1, the most important value (0.91 ± 0.02) being recorded in the case of variant V3-8.5 with 8.50% vol. alcohol. Excluding acetic acid, all the identified organic acids were found in the highest amount in the ROVC V3-8.5 and TSVC V1-3.5 samples; for malic acid, the differences between these two variants were non-significant. Compared to the base wine (control), the TSVC V3-8.5 sample showed non-significant differences in tartaric, malic, lactic, and acetic acid content.

The concentrations of the main organic acids in the experimental LAW were consistent with values previously reported for white wines [67,75]. For ROVC wines, organic acid levels were comparable to those determined in LAW produced from Muscat Ottonel grape must by reverse osmosis followed by subsequent fermentation [76].

### 3.7. Analysis of Volatile Compounds in LAW

Aroma represents a key indicator in the evaluation of wine quality, directly influencing consumer preferences; moreover, its complexity and balance play a decisive role in the market acceptance of wine [77]. Wine aroma and taste are critical determinants of consumer acceptance, with volatile compounds representing the primary contributors [78,79]. The type and concentration of these compounds, their sensory thresholds, and the interactions among them collectively define the flavor profile and typicity of wines. However, wine aroma is influenced by multiple factors, including grape variety, climatic conditions, viticultural practices, fermentation parameters, microbial flora, and production technology [80,81,82].

**Figure 5:**
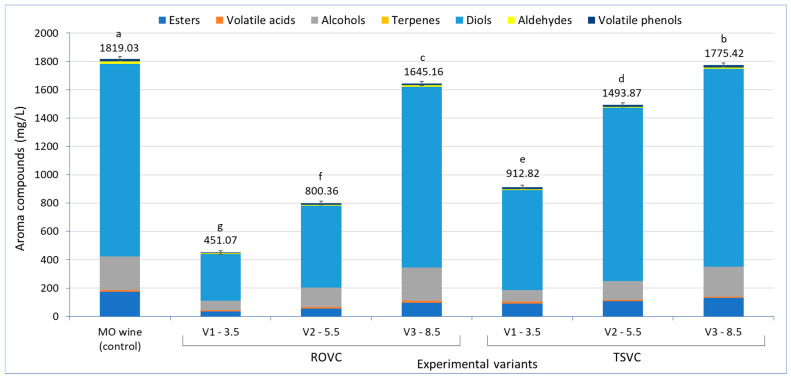
Volatile fractions of Muscat Ottonel low-alcohol wines obtained by reverse osmosis-vacuum concentration (ROVC) and two-step vacuum concentration (TSVC). Different letters indicate significant differences between the mean values in Tukey’s multiple range test (*p* < 0.05).

The aroma of wine is shaped by a complex mixture of volatile compounds, with alcohols, esters, aldehydes, ketones, lactones, acids, terpenes, phenols, and sulphur compounds being the most significant contributors [82,83]. More than 1000 volatile compounds have been found in wines, with a total content that reaches approximately 0.8–1.2 g/L, coming from a variety of sources (raw material, fermentations, or aging stage) [81,84,85,86,87]. In Muscat Ottonel LAW produced by the two technologies, a total of 25 aroma compounds were identified and quantified, being classified in seven chemical classes, as follows: five esters, three volatile fatty acids, nine alcohols, one terpene, two diols, two aldehydes, and three volatile phenols. Considering the total concentration of each group of compounds, the order was similar for all analyzed wines: diols > alcohols > esters > aldehydes > acids > phenols > terpenes. A comparison of volatile compounds in the LAW obtained by the two processes is shown in Figure 5.

Analysis of the total volatile compound content revealed significant differences among the wine samples produced by the two processes. In the control wine, the total volatile compound content was 1819.03 mg/L. Despite the significant differences observed, wines with an alcoholic strength of 8.50% vol. (V3-8.5), obtained by both processes, showed comparable values (1645.16 and 1775.40 mg/L). Overall, the total volatile compound content decreased proportionally with the alcoholic strength of the samples. At similar alcoholic strength, the lowest total volatile compound contents were observed in LAW produced by the reverse osmosis-vacuum concentration (ROVC) process.

Among the ROVC samples, the V3-8.5 variant exhibited the highest concentrations of alcohols, aldehydes, and volatile acids (233.44, 12.87, and 14.09 mg/L, respectively). The volatile acid content exceeded that of the control wine (12.96 mg/L), an outcome attributed to their concentration in the reverse osmosis retentate.

In the TSVC V3-8.5 wine, esters, terpenes, diols, and volatile phenols (131.35, 210.98, 0.31, 1397.52, and 18.76 mg/L, respectively) were present in the highest concentration among the analyzed wines.

Esters are the largest family of volatile compounds in wine, an important contributor to fruity aroma [88,89]. Wines obtained by the TSVC process presented a total ester content of two- (V2-5.5) and up to three-fold (V1-3.5) higher compared to wines obtained through ROVC. The TSVC V3-8.5 sample showed a total ester content reduced by up to 24% relative to the control wine. Although dealcoholization of wine by membrane processes has been reported to significantly affect its volatile profile, depending on the technique employed and the degree of alcohol reduction [23,32], Sam et al. [14] demonstrated that reverse osmosis may retain more esters than vacuum distillation due to lower operating temperature. In the present study, however, this potential advantage was offset because the retentate obtained by reverse osmosis was subsequently diluted with alcohol-free water.

Although the aldehyde content remained at low levels (<18 mg/L), the ROVC wines showed higher concentrations of aldehydes (up to 12.87 mg/L in V3-8.5) compared to TSVC wines (7.03 mg/L in V3-8.5). Aldehydes are volatile compounds that influence the organoleptic properties of wine. Many changes in wine aroma linked to oxidation are very often related to the formation of aldehydes [90]. Comparable concentrations of aroma compounds were reported in Muscat de Alexandria wines [80] and in Muscat Ottonel wines [81].

**Figure 6:**
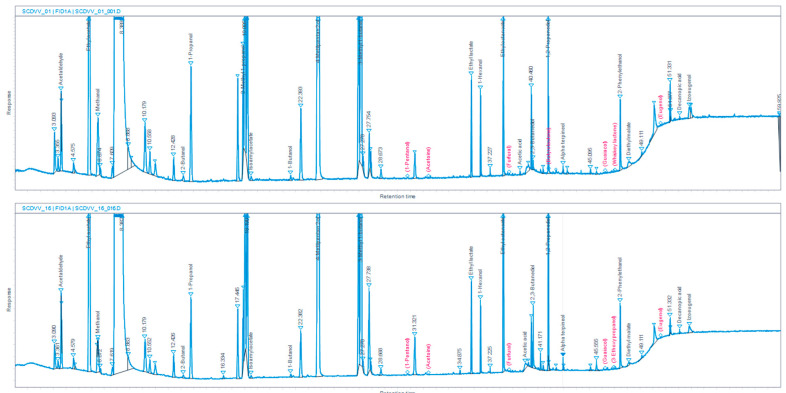
GC-chromatograms of volatile compounds identified in Muscat Ottonel low-alcoholic wines.

Based on the chromatograms obtained by gas chromatography (Figure 6), individual concentrations of aroma compounds identified in LAWs produced by the two tested processes are summarized in Table 6.

In LAWs, higher alcohols were the most diverse group of volatile compounds, with 3-methyl-1-butanol (isoamyl alcohol) as the most abundant (32.57–125.70 mg/L). Higher alcohols have an important impact on the wine fruity aroma [36]. In the ROVC wines, the concentration of volatile alcohols varied significantly, the values increasing along with alcohol concentration. In LAW with 8.5% vol. alcohol (V3-8.5) obtained by ROVC, 2-butanol (0.15 mg/L), 2-methyl-1-butanol (amyl alcohol) (27.22 mg/L), and 3-methyl-1-butanol (isoamyl alcohol) (125.70 mg/L) were non-significantly different from the control wine. However, 3-ethoxy-1-propanol was not detected in any of the ROVC samples.

Polyalcohols (polyols) influence more the taste and less the smell of the wines, these becoming smoother and more full-bodied [94]. By introducing the second hydroxyl group into the molecule (diols), the characteristic alcohol smell of monohydroxy alcohols disappears and the sweet, sometimes bittersweet taste of polyols appears. 2,3-butanediol, formed during alcoholic fermentation by the reduction in acetoin, and known to impart creamy and buttery notes to wine [36,95], was identified at the highest concentration among all volatile compounds identified (250.39–742.25 mg/L), with higher values observed in ROVC samples with higher alcohol strength.

At high dilutions of wine concentrated by RO, some aldehydes, such as furfural or volatile phenols (guaiacol, eugenol), were no longer detected in LAW with 3.50% vol. alcohol (ROVC V1-3.5).

As previously reported, fruity aroma is closely associated with the presence of esters and higher alcohols [36,81,96]. In the ROVC experimental wines, diethyl malate, ethyl acetate, and ethyl lactate were the most abundant esters, with concentrations ranging from 40.77 to 62.66 mg/L in the control wine. These levels decreased significantly in LAW obtained by reverse osmosis due to the addition of osmosis water. In the 8.5% vol. alcohol variant (ROVC V3-8.5) ethyl lactate was present in higher concentrations than in the control wine. However, isoamyl acetate, diethyl succinate, and diethyl malate were no longer found in wines with low alcohol concentration obtained by reverse osmosis-vacuum concentration (ROVC V1-3.5).

Terpenes are varietal compounds essentially coming from grapes as enzymatically produced secondary metabolites of the terpenoid pathway [47,97]. Terpenes play a prominent role in the floral character of Muscat wines, in small concentration acting as enhancers of aroma, adding complexity to the wine. According to Blagoeva et al. [98] α-terpineol, as one of the main terpene compounds in Muscat Ottonel wines, can be found in concentrations of 0.30–0.50 mg/L, giving aroma notes of lilac and melon. In LAW obtained by ROVC α-terpineol ranged from 0.14 (V1-3.5) to 0.26 (V3-8.5) mg/L, non-significant differences were recorded between wine samples with 3.5% vol. (V1) and 5.5% vol. (V2) alcohol. These values are low, affecting the aromatic complexity of LAW. However, the retention of aroma compounds in retentate is influenced by membrane properties, molecular weight, volatility, activity coefficient of each compound, and the non-volatile matrix of the wine [14,99].

Unlike the LAW obtained by ROVC, LAW obtained by applying vacuum at low temperature (TSVC) showed lower variations in volatile compounds due to the addition of smaller volumes of the aromatic fraction F1 in the concentrated wine. However, the differences between LAW samples with different alcohol concentrations were mostly significant. With the exception of eugenol (volatile phenol), in the sample with 3.5% vol. alcohol (TSVC V1-3.5), all the aroma compounds identified in the control wine Muscat Ottonel could be quantified. The LAW with 8.5% vol. alcohol (TSVC V3-8.5) showed abundant amounts of volatile compounds, close to those found in the control wine or even higher in the case of 2-phenylethanol, 3-ethoxy-1-propanol, 1-hexanol (alcohols), 1,2-propanediol (diol), ethyl lactate (ester), furfural (aldehyde), guaiacol, and isoeugenol (volatile phenols) (Table 6) due to their extraction during the first step of vacuum concentration, specifically in fraction F1, which was subsequently used for the alcoholization of the concentrated wine.

In wines obtained through TSVC, α-terpineol was found in higher amount in samples with alcohol above 5.50% vol. (V2-5.5 and V3-8.5), the differences being significant compared to the LAW with similar alcohol concentrations obtained through ROVC. Moreover, in TSVC samples volatile acids (hexanoic, octanoic, and decanoic acids) and some alcohols (like 2-phenylethanol) showed an inverse trend with the increase in alcohol concentration. Thus, wines with lower alcohol concentrations showed higher amounts of volatile acids (rancid, fatty, waxy sensation at higher concentrations) and 2-phenylethanol (rose-like, floral aroma). Fatty acids are short and medium carbon-chained organic acids produced from lipid metabolism, with lower volatility and limited solubility in water [100]; thus, hexanoic acid is partially volatilized, but octanoic and decanoic acids can remain in larger quantities in concentrated wine, increasing the risk of unpleasant sensory notes. At the same time, in smaller concentrations, acids such as hexanoic, octanoic, and decanoic are relevant for the formation of the relative esters, which can give pleasant and fruity/floral character to the wine [100,101]. A similar situation was found in the case of 2-phenylethanol, which has low volatility and remains largely in the concentrated wine matrix, accentuating the floral notes [101,102].

Comparing the LAW obtained by the two tested methods, it was observed that in the case of reverse osmosis and retentate dilution (ROVC) the reduction in the alcoholic strength resulted in a greater loss of volatile compounds, especially esters, some volatile acids (decanoic acid), most of the alcohols, and terpenes, significantly affecting the aromatic profile of LAW (V3-3.5). In the case of wine vacuum concentration (TSVC), the losses of aroma compounds (excepting 1-propanol, 2-methyl-1-butanol, and 3-methyl-1-butanol) were lower compared to reverse osmosis.

Previous studies have shown that both methods may lead to the loss of volatile compounds such as ethyl esters and aliphatic alcohols [14,31]. Except for samples with 3.50% vol. alcohol, the concentrations of volatile compounds in LAW obtained by the two procedures were comparable to those reported for various white wines [80,88,101,103].

### 3.8. Sensorial Evaluation of LAW

**Figure 7:**
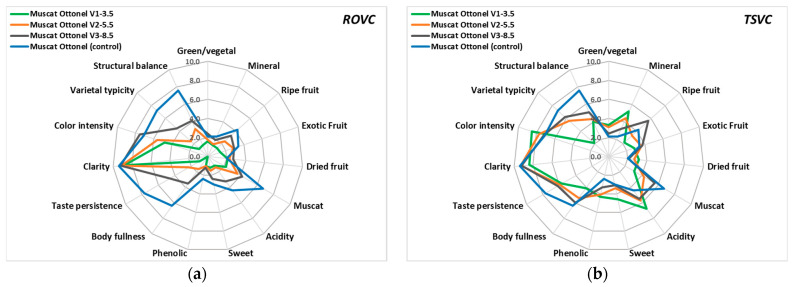
Sensory analysis of Muscat Ottonel LAW obtained through (**a**) reverse osmosis-vacuum concentration ROVC and (**b**) two-step vacuum concentration TSVC. Note: A 10-point scale, where 1 indicates not perceptible and 10 indicates extremely intense sensation. Average values of the scores given by the tasters (*n* = 9).

Wine quality is closely linked to sensory attributes such as aroma, taste, and mouthfeel [104]. The perception of wine flavor and aroma is the result of a multitude of interactions between a large number of chemical compounds and sensory receptors [94]. For the sensory analysis of the experimental wines, 15 characteristics were assessed by a panel of tasters using a structured scale ranging from 1 (lowest perception) to 10 (highest perception). Muscat Ottonel LAW obtained by ROVC appeared clear, comparable to the control wine, with a total acidity and muscat/ripe fruit aromas decreasing as alcoholic strength was reduced (Figure 7a). Compared to the control wine, LAW with 8.50% vol. ethanol (ROVC V3-8.5) showed subtle fruity aroma (3.3), a low phenolic perception (1.2), and slightly darker color (7.5), probably due to oxidation with the addition of osmosis water. Also, taste persistence (4.6), body fullness (3.5), structural balance (4.1), and varietal typicity (4.4) were all rated lower than those observed in the control wine. The ROVC V1-3.5 sample, with the lowest alcohol content (3.50% vol. ethanol), was characterized by a more reduced aromatic complex (faint muscat aroma) (2.2), weaker acidity (1.2), and a lower color intensity (lighter color) (4.8).

In parallel, sensory evaluation of TSVC V3-8.5 wine (8.50% vol. ethanol) indicated a more intense fruity aroma (3.7–5.6), arising from the combined influence of volatile compounds such as acetates, ethyl esters, and higher alcohols [105], a muscat aroma (terpenes) very close to the control wine (5.6), a good body-fullness (6.0), and higher varietal typicity (6.2) and color intensity (7.2) (Figure 7b). On the other side, taste persistence (6.2) was lower compared to the conventional Muscat Ottonel wine (control) (7.7). As the alcoholic strength decreased, the LAW produced by TSVC displayed a more pronounced mineral character and elevated acidity, attributable to the reduced dilution of the wine with the aromatic fraction (F1). This, in turn, diminished the varietal typicity of the wine, as the aroma profile became less complex. Thus, samples with 3.5% vol. alcohol obtained by TSVC (V1-3.5) showed a higher acidity (6.8), a stronger mineral character (5.2) and sweetness (4.6), a weak dried fruit aroma (possible due to the presence of higher concentrations of volatile acids and aldehydes) (3.2), good taste persistence (5.7), and structural balance (4.0). However, all TSVC-derived LAWs were perceived as more closely aligned with the varietal character, in contrast to those produced by reverse osmosis and subsequent dilution.

As indicated in previous studies, ethanol concentration significantly affects sensory perception of aroma compounds in wine. According to De-la-Fuente-Blanco et al. [106], at alcohol levels below 7.5% white wine may become unbalanced, and at high ethanol levels becomes more full bodied and its aroma is more intense. Also, an increase in ethanol concentration can decrease the intensity of fruity and floral aromas [106,107]. Moreover, applying vacuum distillation in white wines resulted in higher color intensity of the dealcoholized wines, while reverse osmosis caused notable color differences between LAW samples [108]. Overall, although samples with similar alcoholic strength maintained some sensory similarity, their organoleptic expression was strongly shaped by the applied production technology. Due to the loss of key esters, such as ethyl acetate and isoamyl acetate, essential for enhancing aroma perception, dealcoholized wines generally show diminished aroma, flavor balance, and overall sensory quality [22,99].

### 3.9. Statistical Analysis

**Figure 8:**
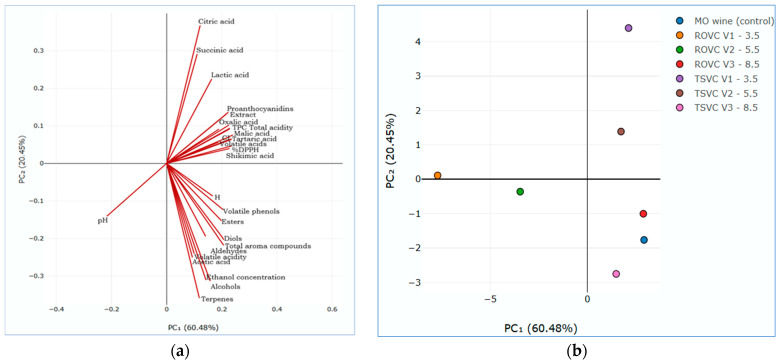
Principal component analysis (PCA) biplot combining (**a**) the output variables and (**b**) grouping the low-alcohol wine variants.

Principal component analysis (PCA) is a multivariate technique that allows visual representation of the correlations between multiple variables, increasing data interpretability and explaining data variation [1]. PCA biplot indicates how the vectors (physico-chemical features, phenolic content, antioxidant activity, chromatic parameters, organic acid and volatile profile) are distributed (Figure 8a) and groups LAW variants according to the first two principal components, PC1 (60.48%) and PC2 (20,45%) (Figure 8b). The red lines indicate how each variable correlates with the principal components. Lines oriented in the same direction are positively correlated, while the longer a line, the more that variable contributes to the variability explained by PCA. In Figure 8b, points close to each other indicate LAW variants with similar chemical and sensory characteristics. By overlaying the two plots (Figure 8a,b), the location of the points (variants) relative to the red lines (variables) indicates which characteristics influence each experimental LAW. The PCA figure indicated that LAW variants can be grouped according to their physico-chemical and organoleptic characteristics, and these groupings can guide selection for different types of consumers or applications (sensory preferences). Thus, based on the distribution of points and vectors, a first group characterized by greater aromatic richness and higher alcohol strength can be distinguished. This group is oriented toward esters, terpenes, and alcoholic concentration and comprises the control wine together with both 8.50% vol. alcohol variants (ROVC V3-8.5 and TSVC V3-8.5). The second group, positioned in the direction of acids (citric, succinic lactic), volatile acids, phenolic compounds, and antioxidant activity, characterized by a higher acidity, includes the variants with 5.50 and 3.50% vol. alcohol obtained through TSVC (V1-3.5 and V2-5.5). The third group, strongly influenced by pH, weakly aromatic, covers the two LAW variants obtained through ROVC (V1-3.5 and V2-5.5).

**Figure 9:**
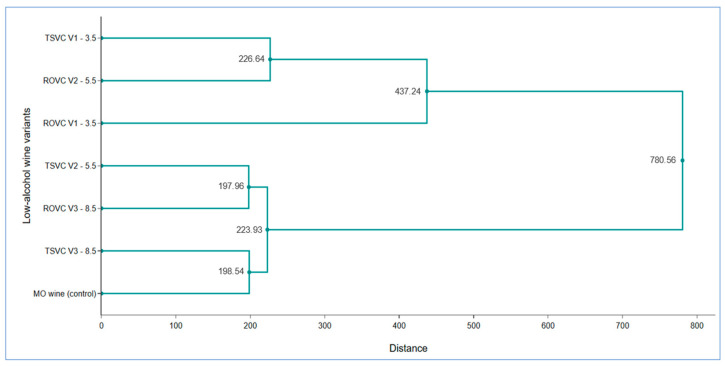
Hierarchical cluster analysis of the Muscat Ottonel LAW obtained through reverse osmosis-vacuum concentration ROVC and two-step vacuum concentration TSVC.

The cluster method admits the existence of the polythetic groups, whose elements are equivalent or similar for most criteria, measuring the similitude of the elements from the group and the difference among groups. However, cluster analysis is a convenient method for identifying homogenous groups considering a multitude of factors. Agglomerative Hierarchical Clustering (AHC), single-linkage (Euclidian distance), was used to group the LAW samples, considering all the features analyzed in the study. Thus, the hierarchical cluster analysis indicated the formation of two main clusters, coupled at great distances in a common node (Figure 9).

The first cluster includes two homogeneous subgroups, one formed by linking the control wine with the TSVC V3-8.5 (8.50% vol.) variant, and the second formed by TSVC V2-5.5 (5.50% vol.) and ROVC V3-8.5 (8.50% vol.) variants. Subgroups were closely positioned (197.96–198.54), with aggregation yielding a higher distance (223.93). The second homogenous cluster was formed by ROVC V2-5.5 (5.50% vol.) and TSVC V1-3.5 (3.50% vol.), to which the LAW ROVC V1-3.5 (3.50% vol.) variant is also attached at a greater distance (437.24), indicating partial similarity of the attached variants.

## 4. Conclusions

In a dynamic wine market, alcohol management remains a major challenge for producers. The dealcoholization process must be carefully selected and fully understood, aiming at preserving wine quality while reducing ethanol content. In this context, two partial alcohol reduction techniques were evaluated: (i) reverse osmosis coupled with vacuum concentration of the hydroalcoholic permeate, followed by controlled blending of the retentate with osmosis water (ROVC), and (ii) vacuum concentration of Muscat Ottonel wine with subsequent controlled blending of the primary alcoholic–aromatic fraction with the alcohol-free wine (TSVC). The dilution applied in the first process (ROVC) was intended to reduce the alcoholic strength of concentrated wine, whereas in the second process (TSVC) the dilution was employed to increase the alcoholic strength while simultaneously restoring the aromatic fraction. Beverages with alcoholic concentrations of 3.50, 5.50, and 8.50% vol. were obtained, displaying compositional characteristics and sensory properties that varied significantly according to both the final alcoholic strength and the dealcoholization technique applied. ROVC yielded wines in which phenolic compounds, organic acids, volatile constituents, and color intensity decreased progressively with the reduction in alcohol concentration. Thus, ROVC wines with 3.50% vol. alcohol were characterized by the lowest levels of extract, organic acids, and volatile compounds, together with a diminished aromatic complexity, weak acidity, and lighter color resulting from dilution with osmosis water. Compared to reverse osmosis, TSVC produced low-alcohol wines with significantly higher phenolic content and antioxidant activity, and elevated concentrations of volatile compounds (esters and terpenes), thereby better preserving the typicity of wines as confirmed through sensory evaluation. In TSVC, wines with lower alcohol content exhibited higher values of chromatic parameters and organic acids, an inverse trend compared to ROVC, attributable to the reduced dilution of concentrated wine. The results demonstrated that both technologies tested represent promising approaches for partial dealcoholization of wine, with two-step vacuum concentration emerging as essential processes for preserving aromatic complexity. Low-alcohol beverages obtained through TSVC were statistically closer to conventional wine, as the use of low temperature allowed the recovery of most volatile compounds and their reintegration into the concentrated wine, thereby minimizing the impact on taste and overall quality. However, even when applying TSVC, further reduction in alcoholic strength below 5.50% vol. may significantly compromise the quality and acceptability of the final product. These findings improve the understanding of how the extent of dealcoholization and the applied technologies affect the composition and perception of wine and provide valuable insights for small and medium-sized producers aiming to obtain low-alcohol wines with minimal quality loss. By integrating these technologies, the winemakers can successfully respond to current consumer demands while enhancing product quality, sustainability, and operational efficiency.

## Figures and Tables

**Table 1:** Physico-chemical characterization of the initial Muscat Ottonel wine and fractions resulting from the RO process.

Sample	Volume (L)	Alcoholic Concentration (% vol.)	Total Acidity (g/L as Tartaric Acid)	Volatile Acidity (g/L as Acetic Acid)	Free SO_2_ (mg/L)	Total SO_2_ (mg/L)	Reducing Sugars (g/L)	pH
Initial wine (Control)	300	11.80 ± 0.05 ^b^	4.82 ± 0.02 ^b^	0.42 ± 0.02 ^b^	12.24 ± 0.42 ^b^	58.00 ± 0.62 ^b^	0.90 ± 0.07 ^b^	3.49 ± 0.02 ^b^
Concentrated wine (Retentate)	150	12.85 ± 0.05 ^a^	7.78 ± 0.03 ^a^	0.65 ± 0.02 ^a^	21.40 ± 0.28 ^a^	99.00 ± 0.81 ^a^	1.76 ± 0.04 ^a^	3.11 ± 0.02 ^c^
Hydroalcoholic fraction (Permeate)	150	10.72 ± 0.05 ^c^	1.85 ± 0.03 ^c^	0.16 ± 0.01 ^c^	2.60 ± 0.13 ^c^	4.20 ± 0.11 ^c^	0.01 ± 0.00 ^c^	4.42 ± 0.02 ^a^

Note: Data presented as mean values with standard deviation (±). Different letters on the same column indicate significant differences between the mean values in Tukey’s multiple range test.

**Table 2:** Physico-chemical characteristics of the experimental low-alcohol wine obtained by reverse osmosis-vacuum concentration (ROVC) and two-step vacuum concentration (TSVC).

Features	MO Wine (Control)	ROVC			TSVC		
		V1-3.5	V2-5.5	V3-8.5	V1-3.5	V2-5.5	V3-8.5
Density at 20 °C (g/cm^3^)	0.9911 ± 0.0001 ^g^	0.9969 ± 0.0001 ^c^	0.9945 ± 0.0001 ^d^	0.9932 ± 0.0001 ^f^	1.0030 ± 0.0001 ^a^	0.9975 ± 0.0001 ^b^	0.9938 ± 0.0001 ^e^
Alcoholic concentration (% vol.)	11.80 ± 0.02 ^a^	3.50 ± 0.02 ^d^	5.50 ± 0.02 ^c^	8.50 ± 0.02 ^b^	3.50 ± 0.02 ^d^	5.50 ± 0.02 ^c^	8.50 ± 0.02 ^b^
Total acidity (g/L as tartaric acid)	4.82 ± 0.05 ^d^	2.55 ± 0.04 ^f^	3.60 ± 0.08 ^e^	4.80 ± 0.06 ^d^	5.47 ± 0.08 ^a^	5.25 ± 0.09 ^b^	4.95 ± 0.04 ^c^
Volatile acidity (g/L as acetic acid)	0.42 ± 0.04 ^c^	0.34 ± 0.02 ^d^	0.58 ± 0.04 ^b^	0.82 ± 0.08 ^a^	0.38 ± 0.03 ^cd^	0.62 ± 0.05 ^b^	0.96 ± 0.06 ^a^
Free SO_2_ (mg/L)	12.24 ± 0.06 ^d^	11.21 ± 0.04 ^e^	25.14 ± 0.08 ^b^	33.37 ± 0.06 ^a^	5.11 ± 0.06 ^g^	8.29 ± 0.07 ^f^	12.08 ± 0.08 ^c^
Total SO_2_ (mg/L)	52.00 ± 0.18 ^c^	28.68 ± 0.30 ^g^	67.11 ± 0.38 ^b^	88.65 ± 0.32 ^a^	39.27 ± 0.41 ^f^	41.00 ± 0.30 ^e^	48.10 ± 0.37 ^d^
Reducing sugars (g/L)	0.90 ± 0.04 ^c^	0.18 ± 0.02 ^e^	0.65 ± 0.06 ^d^	0.98 ± 0.08 ^bc^	1.21 ± 0.06 ^a^	1.06 ± 0.04 ^b^	1.00 ± 0.06 ^b^
Non-reducing extract (g/L)	21.00 ± 0.30 ^b^	9.32 ± 0.30 ^f^	9.95 ± 0.30 ^e^	16.24 ± 0.30 ^d^	24.29 ± 0.30 ^a^	17.24 ± 0.30 ^c^	17.50 ± 0.30 ^c^
pH	3.49 ± 0.02 ^b^	3.61 ± 0.02 ^a^	3.58 ± 0.02 ^a^	3.46 ± 0.02 ^bc^	3.40 ± 0.02 ^c^	3.42 ± 0.03 ^c^	3.45 ± 0.02 ^bc^

Note: Data presented as mean values with standard deviation (±) (*n* = 3). Different letters on the same row indicate significant differences between the mean values in Tukey’s multiple range test (*p* < 0.05).

**Table 3:** Polyphenolic content and antioxidant activity of the experimental low-alcohol wines.

Sample		TPC (g GAE/L)	TPI (OD at 280 nm)	Proanthocyanidins (g CE/L)	% Scavenged DPPH*
MO wine (control)		0.36 ± 0.02 ^b^	6.41 ± 0.02 ^d^	0.13 ± 0.01 ^b^	58.69 ± 0.49 ^c^
ROVC	V1-3.50	0.19 ± 0.01 ^d^	3.72 ± 0.01 ^f^	0.04 ± 0.01 ^e^	30.01 ± 0.28 ^e^
	V2-5.50	0.28 ± 0.02 ^c^	4.59 ± 0.02 ^e^	0.08 ± 0.02 ^d^	38.92 ± 0.39 ^d^
	V3-8.50	0.41 ± 0.03 ^a^	7.43 ± 0.02 ^b^	0.16 ± 0.02 ^c^	61.06 ± 0.40 ^b^
TSVC	V1-3.50	0.44 ± 0.01 ^a^	7.78 ± 0.04 ^a^	0.19 ± 0.02 ^a^	62.46 ± 0.48 ^a^
	V2-5.50	0.42 ± 0.02 ^a^	7.70 ± 0.04 ^a^	0.17 ± 0.02 ^a^	61.11 ± 0.50 ^b^
	V3-8.50	0.39 ± 0.02 ^ab^	7.22 ± 0.03 ^c^	0.15 ± 0.02 ^ab^	59.08 ± 0.41 ^c^

Note: ROVC—reverse osmosis-vacuum concentration; TSVC—two-step vacuum concentration; TPC—total phenolic content; GAE—gallic acid equivalent; TPI—total phenolic index; OD280 nm—optical density at 280 nm; CE—catechin equivalent; DPPH—2,2-diphenyl-1-picrylhydrazyl free radical. Data presented as mean values with standard deviation (±) (*n* = 3). Different letters on the same column indicate significant differences between the mean values in Tukey’s multiple range test (*p* < 0.05).

**Table 4:** Chromatic parameters of experimental low-alcohol wines obtained by reverse osmosis-vacuum concentration (ROVC) and two-step vacuum concentration (TSVC).

Sample		%Y	%R	%B	CI	H
MO wine (control)		78.04 ± 0.02 ^a^	19.75 ± 0.02 ^f^	2.21 ± 0.02 ^g^	0.07 ± 0.02 ^b^	3.95 ± 0.01 ^a^
ROVC	V1-3.50	69.47 ± 0.02 ^g^	23.01 ± 0.03 ^a^	7.52 ± 0.04 ^c^	0.02 ± 0.01 ^d^	3.02 ± 0.01 ^f^
	V2-5.50	70.70 ± 0.03 ^f^	21.03 ± 0.02 ^d^	8.28 ± 0.03 ^b^	0.06 ± 0.02 ^bc^	3.36 ± 0.01 ^d^
	V3-8.50	70.99 ± 0.02 ^e^	20.63 ± 0.01 ^e^	8.38 ± 0.02 ^a^	0.11 ± 0.02 ^a^	3.44 ± 0.01 ^b^
TSVC	V1-3.50	71.62 ± 0.03 ^b^	21.01 ± 0.02 ^d^	7.37 ± 0.01 ^d^	0.10 ± 0.03 ^a^	3.41 ± 0.01 ^c^
	V2-5.50	71.49 ± 0.02 ^c^	21.38 ± 0.03 ^c^	7.13 ± 0.01 ^e^	0.09 ± 0.03 ^ab^	3.34 ± 0.01 ^d^
	V3-8.50	71.38 ± 0.02 ^d^	21.61 ± 0.02 ^b^	7.01 ± 0.01 ^f^	0.09 ± 0.02 ^ab^	3.30 ± 0.01 ^e^

Note: %Y—proportion of yellow; %R—proportion of red; %B—proportion of blue; CI—color intensity; H—color hue. Data presented as mean values with standard deviation (±) (*n* = 3). Different letters within the same column indicate significant differences between the mean values in Tukey’s multiple range test (*p* < 0.05).

**Table 5:** Organic acid profile of experimental Muscat Ottonel low-alcohol wines.

Acids	MO Wine (Control)	ROVC			TSVC		
		V1-3.5	V2-5.5	V3-8.5	V1-3.5	V2-5.5	V3-8.5
Tartaric acid (g/L)	4.31 ± 0.02 ^a^	1.16 ± 0.02 ^e^	2.51 ± 0.08 ^d^	3.81 ± 0.03 ^b^	4.37 ± 0.05 ^a^	3.77 ± 0.02 ^b^	3.53 ± 0.07 ^c^
Malic acid (g/L)	0.89 ± 0.02 ^a^	0.29 ± 0.01 ^e^	0.60 ± 0.04 ^d^	0.93 ± 0.03 ^a^	0.92 ± 0.06 ^a^	0.83 ± 0.02 ^b^	0.70 ± 0.04 ^c^
Lactic acid (g/L)	0.82 ± 0.03 ^a^	0.45 ± 0.02 ^d^	0.48 ± 0.03 ^d^	0.58 ± 0.04 ^c^	0.84 ± 0.03 ^a^	0.79 ± 0.03 ^b^	0.48 ± 0.02 ^d^
Citric acid (g/L)	0.55 ± 0.02 ^c^	0.42 ± 0.04 ^d^	0.53 ± 0.01 ^c^	0.59 ± 0.03 ^c^	1.04 ± 0.02 ^a^	0.78 ± 0.04 ^b^	0.49 ± 0.03 ^cd^
Acetic acid (g/L)	0.38 ± 0.03 ^d^	0.32 ± 0.01 ^e^	0.53 ± 0.03 ^c^	0.78 ± 0.02 ^b^	0.33 ± 0.02 ^de^	0.56 ± 0.04 ^c^	0.91 ± 0.02 ^a^
Oxalic acid (mg/L)	67.92 ± 0.08 ^f^	33.20 ± 0.16 ^g^	73.97 ± 0.12 ^e^	128.15 ± 0.15 ^a^	101.90 ± 0.62 ^b^	93.91 ± 0.29 ^c^	78.47 ± 0.33 ^d^
Succinic acid (mg/L)	539.58 ± 0.17 ^b^	425.71 ± 0.09 ^f^	452.81 ± 0.11 ^e^	490.31 ± 0.17 ^d^	580.41 ± 0.11 ^a^	533.16 ± 0.14 ^c^	356.44 ± 0.31 ^g^
Shikimic acid (mg/L)	9.43 ± 0.07 ^d^	4.34 ± 0.04 ^f^	7.74 ± 0.06 ^e^	12.54 ± 0.05 ^a^	10.81 ± 0.07 ^b^	9.76 ± 0.11 ^c^	9.46 ± 0.09 ^d^

Note: ROVC—reverse osmosis-vacuum concentration; TSVC—two-step vacuum concentration. Data presented as mean values with standard deviation (±) (*n* = 3). Different letters within the same row indicate significant differences between the mean values in Tukey’s multiple range test (*p* < 0.05).

**Table 6:** Volatile compounds (mg/L) in Muscat Ottonel LAW obtained by reverse osmosis-vacuum concentration (ROVC) and two-step vacuum concentration (TSVC).

No.	Chemical Category	Compound (mg/L)	MO Wine (Control)	ROVC			TSVC			Olfactory Descriptor [81,91,92,93]
				V1-3.5	V2-5.5	V3-8.5	V1-3.5	V2-5.5	V3-8.5	
1	Esters	Ethyl acetate	57.45 ± 1.08 ^a^	6.62 ± 0.53 ^e^	14.55 ± 0.74 ^d^	31.29 ± 0.43 ^b^	7.97 ± 0.21 ^e^	15.35 ± 0.19 ^d^	28.81 ± 0.17 ^c^	Fruity, sweet
2		Isoamyl acetate	9.83 ± 0.06 ^a^	n.d.	2.81 ± 0.15 ^d^	4.01 ± 0.09 ^c^	0.21 ± 0.02 ^e^	4.01 ± 0.04 ^c^	6.01 ± 0.09 ^b^	Banana, pear
3		Ethyl lactate	40.77 ± 0.54 ^d^	29.52 ± 0.27 ^f^	37.70 ± 0.32 ^e^	55.20 ± 0.51 ^a^	43.15 ± 0.21 ^c^	44.50 ± 0.43 ^b^	45.31 ± 0.41 ^b^	Creamy, buttery
4		Diethyl succinate	3.13 ± 0.08 ^a^	n.d.	1.69 ± 0.03 ^d^	3.17 ± 0.09 ^a^	1.24 ± 0.06 ^e^	2.06 ± 0.04 ^c^	2.46 ± 0.11 ^b^	Fruity, mild
5		Diethyl malate	62.66 ± 0.19 ^a^	n.d.	0.09 ± 0.01 ^f^	3.00 ± 0.10 ^e^	39.23 ± 0.61 ^d^	41.27 ± 0.42 ^c^	48.76 ± 0.47 ^b^	Apple-like, soft fruit
6	Volatile acids	Hexanoic acid	1.49 ± 0.02 ^b^	0.91 ± 0.05 ^e^	1.13 ± 0.02 ^d^	1.73 ± 0.05 ^a^	1.32 ± 0.11 ^c^	1.20 ± 0.07 ^d^	1.15 ± 0.04 ^d^	Rancid, cheesy (in excess)
7		Octanoic acid	9.47 ± 0.12 ^c^	6.88 ± 0.13 ^f^	7.57 ± 0.18 ^d^	11.06 ± 0.09 ^a^	10.09 ± 0.21 ^b^	7.30 ± 0.14 ^e^	7.10 ± 0.13 ^ef^	Fatty, waxy
8		Decanoic acid	2.00 ± 0.08 ^a^	0.17 ± 0.02 ^d^	1.08 ± 0.05 ^c^	1.30 ± 0.07 ^b^	1.42 ± 0.08 ^b^	1.38 ± 0.05 ^c^	1.22 ± 0.02 ^d^	Soapy, fatty
9	Alcohols	1-Propanol	25.94 ± 0.21 ^a^	5.29 ± 0.11 ^f^	9.17 ± 0.17 ^d^	17.43 ± 0.09 ^b^	4.92 ± 0.06 ^g^	8.80 ± 0.10 ^e^	16.36 ± 0.07 ^c^	Mild alcoholic, solvent
10		2-Butanol	0.15 ± 0.01 ^a^	0.09 ± 0.02 ^a^	0.13 ± 0.02 ^a^	0.15 ± 0.02 ^a^	0.12 ± 0.02 ^a^	0.12 ± 0.03 ^a^	0.14 ± 0.01 ^a^	Slightly sweet, alcoholic
11		1-Butanol	0.66 ± 0.04 ^a^	0.18 ± 0.06 ^d^	0.27 ± 0.02 ^c^	0.48 ± 0.07 ^b^	0.15 ± 0.02 ^d^	0.28 ± 0.02 ^c^	0.46 ± 0.04 ^b^	Herbaceous, “green”
12		2-Methyl- 1-propanol	33.45 ± 0.26 ^a^	1.48 ± 0.09 ^g^	16.46 ± 0.14 ^d^	32.18 ± 0.30 ^b^	8.16 ± 0.26 ^f^	15.58 ± 0.19 ^e^	28.39 ± 0.11 ^c^	Fusel, fermented
13		2-Methyl- 1-butanol	27.53 ± 0.18 ^a^	7.31 ± 0.07 ^e^	15.20 ± 0.10 ^c^	27.22 ± 0.16 ^a^	7.09 ± 0.10 ^f^	14.38 ± 0.12 ^d^	25.78 ± 0.19 ^b^	Fruity, fermented
14		3-Methyl- 1-butanol	126.55 ± 0.29 ^a^	35.26 ± 0.27 ^e^	71.09 ± 0.31 ^c^	125.70 ± 0.59 ^a^	32.57 ± 0.21 ^f^	65.43 ± 0.19 ^d^	116.81 ± 0.23 ^b^	Banana-like, fusel
15		1-Hexanol	1.81 ± 0.03 ^a^	0.64 ± 0.06 ^e^	0.95 ± 0.04 ^d^	1.55 ± 0.02 ^b^	0.51 ± 0.08 ^e^	1.06 ± 0.03 ^c^	1.89 ± 0.07 ^a^	Green, grassy
16		2-Phenylethanol	18.17 ± 0.09 ^f^	15.80 ± 0.31 ^g^	22.70 ± 0.11 ^d^	28.73 ± 0.14 ^a^	26.95 ± 0.27 ^b^	25.02 ± 0.18 ^c^	20.12 ± 0.30 ^e^	Rose-like, floral
17		3-Ethoxy- 1-propanol	0.64 ± 0.04 ^c^	n.d.	n.d.	n.d.	0.57 ± 0.03 ^c^	0.81 ± 0.11 ^b^	1.03 ± 0.02 ^a^	Sweet, ether-like
18	Terpenes	α-terpineol	0.35 ± 0.04 ^a^	0.14 ± 0.02 ^c^	0.16 ± 0.02 ^c^	0.26 ± 0.01 ^b^	0.09 ± 0.01 ^d^	0.18 ± 0.02 ^c^	0.31 ± 0.03 ^a^	Lilac, floral
19	Diols (Polyols)	2,3-Butanediol	904.49 ± 1.21 ^a^	250.39 ± 0.17 ^g^	288.56 ± 0.24 ^f^	742.25 ± 1.31 ^c^	380.35 ± 0.77 ^e^	732.01 ± 1.49 ^d^	797.01 ± 0.95 ^b^	Sweet, viscous mouthfeel
20		1,2-Propanediol	456.45 ± 0.16 ^d^	80.13 ± 0.31 ^g^	289.55 ± 0.52 ^f^	532.97 ± 0.27 ^b^	326.70 ± 0.18 ^e^	491.28 ± 0.42 ^c^	600.51 ± 0.31 ^a^	Mild, solvent-like
21	Aldehydes	Acetaldehyde	17.35 ± 0.15 ^a^	4.32 ± 0.19 ^f^	6.41 ± 0.13 ^c^	12.45 ± 0.11 ^b^	5.63 ± 0.14 ^e^	5.98 ± 0.09 ^d^	6.62 ± 0.09 ^c^	Green apple, pungent
22		Furfural	0.29 ± 0.02 ^c^	n.d.	0.25 ± 0.05 ^c^	0.42 ± 0.09 ^a^	0.25 ± 0.07 ^bc^	0.35 ± 0.04 ^b^	0.41 ± 0.03 ^a^	Almond, baked, caramel
23	Volatile phenols	Guaiacol	1.53 ± 0.02 ^b^	n.d.	0.83 ± 0.09 ^d^	1.54 ± 0.05 ^b^	0.85 ± 0.05 ^d^	1.43 ± 0.02 ^c^	1.91 ± 0.10 ^a^	Smoky, spicy
24		Eugenol	1.48 ± 0.05 ^a^	n.d.	0.11 ± 0.01 ^d^	0.96 ± 0.02 ^b^	n.d.	0.59 ± 0.03 ^c^	0.67 ± 0.06 ^c^	Clove, spicy
25		Isoeugenol	15.39 ± 0.13 ^b^	5.94 ± 0.15 ^f^	10.11 ± 0.12 ^e^	11.90 ± 0.09 ^d^	13.28 ± 0.08 ^c^	13.50 ± 0.21 ^c^	16.18 ± 0.17 ^a^	Floral, spicy

Note: n.d.—not detected. Data presented as mean values with standard deviation (±) (*n* = 3). Different letters within the same row indicate significant differences between the mean values in Tukey’s multiple range test (*p* < 0.05).

## Data Availability

The original contributions presented in this study are included in the article. Further inquiries can be directed to the corresponding author.
